# A cost-minimisation analysis of performing point-of-care ultrasonography on patients with vaginal bleeding in early pregnancy in general practice: a decision analytical model

**DOI:** 10.1186/s12913-022-07463-y

**Published:** 2022-01-11

**Authors:** Swaathi Kiritharan, Mille Vang Johanson, Martin Bach Jensen, Janus Nikolaj Laust Thomsen, Camilla Aakjær Andersen, Cathrine Elgaard Jensen

**Affiliations:** 1 The Faculty of Medicine, Aalborg University, Niels Jernes Vej 10, 9220 Aalborg Øst, Denmark; 2 Center for General Practice, Aalborg University, Fyrkildevej 7, 1,3, 9220 Aalborg Øst, Denmark; 3 Department of Clinical Medicine, Aalborg University, Danish Center for Healthcare Improvements, Fredrik Bajers Vej 5, 176, 9220 Aalborg Øst, Denmark

**Keywords:** Cost Savings, Decision analysis, First Pregnancy Trimester, General practice, Medical economics, Ultrasonography

## Abstract

**Background:**

Spotting and light vaginal bleeding are common and usually harmless symptoms in early pregnancy. Still, vaginal bleeding may be the first sign of an abortion and often causes distress to pregnant women and leads to an expectation of an ultrasonography examination of the uterus. As point-of-care ultrasonography (POCUS) is increasingly being integrated into general practice, these patients may be clinically evaluated and managed by general practitioners (GPs). This can potentially reduce referrals of patients from the primary to the secondary healthcare sector resulting in societal cost-savings.

The primary purpose of this study was to investigate whether the accessibility of POCUS in general practice for patients with vaginal bleeding in early pregnancy is cost-saving compared to usual practice where GPs do not have access to POCUS. A secondary purpose of this study was to estimate a remuneration for GPs performing POCUS on these patients in general practice.

**Methods:**

A cost-minimisation analysis was based on a decision tree model reflecting the two alternatives: general practice with and without GPs having access to POCUS. The robustness of the model results was investigated using probabilistic sensitivity analysis and the following deterministic sensitivity analyses: one-way analyses for the model input parameters and a scenario analysis with a change from a societal to a healthcare sector perspective.

An expected remuneration reflecting the add-on cost of Danish GPs performing POCUS was estimated based on the related costs: cost of an ultrasonography scanner, GP’s time consumption, ultrasonography training, and utensils per scanning.

**Results:**

The difference in average cost between the two alternatives from a societal perspective was estimated to be €110, in favour of general practice with GPs using POCUS. The deterministic sensitivity analyses demonstrated robustness of the results to plausible changes in the input parameters.

The expected remuneration for performing POCUS in this specific setting was estimated to be €32 per examination.

**Conclusion:**

Having GPs perform POCUS on patients with vaginal bleeding in early pregnancy is cost-saving compared to usual practice. The results should be taken with caution as this study was based on early modelling with uncertainties associated with the input parameters in the model.

**Supplementary Information:**

The online version contains supplementary material available at 10.1186/s12913-022-07463-y.

## Background

Over the past decades, ultrasonography devices have become less expensive, smaller, and highly portable [1, 2]. This has enabled point-of-care ultrasonography (POCUS) to be performed in all clinical settings including at the general practitioner (GP), thereby becoming “*the visual stethoscope of the twenty-first century*” [2–5]. By combining ultrasonography findings with GPs’ knowledge of the patient, the patient’s history, and clinical examination, POCUS may allow simple clinical questions to be answered more efficiently within causes originating from different areas of medicine such as the lung, abdomen, or gynaecology and obstetrics [6, 7].

Within the area of gynaecology and obstetrics, GPs can use POCUS in patients with vaginal bleeding for detecting foetal heartbeat in early intrauterine pregnancies to confirm the presence or absence of a viable foetus [8, 9]. Vaginal bleeding is a frequent complication occurring in 20–40% of all pregnancies [9]. The underlying causes for vaginal bleeding can vary widely and originate from intrauterine or extrauterine aetiologies. Though, often a harmless symptom it may also be a sign of ectopic pregnancy, abortion, or trauma [9, 10]. It often causes concern and distress to pregnant women, who seek medical care for the reassurance of a viable pregnancy. In countries where GPs act as gatekeepers for the secondary healthcare sector, e.g. the United Kingdom (UK) and Denmark, patients with vaginal bleeding in early pregnancy will consult their GP for evaluation and they will need a referral from the GP to see a gynaecologist. However, the accessibility and use of POCUS in primary care differs between countries [11, 12]. In countries where POCUS is generally not accessible in general practice, the patients will be referred to a private gynaecological practice or a hospital gynaecology department for further evaluation following a clinical evaluation in general practice [13, 14]. Here transvaginal ultrasonography will be performed [5, 14]. It is, however, believed that the accessibility of POCUS in general practice may potentially enable GPs to provide faster diagnosis and complete the clinical management of many of these patients. Thereby, the number of referrals for this condition to the private gynaecologists and gynaecology departments could potentially be reduced.

The reduction in referrals from GPs to the secondary healthcare sector could lead to cost-savings for the healthcare sector and the patients who avoid time spent on referrals and traveling in addition to faster relief of the concern due to the bleeding episode [14–16]. However, the allocation of performing POCUS from the secondary to the primary healthcare sector must be subject to a remuneration for the GPs to financially be able to provide the service [17]. Currently, GPs in Denmark and several other countries are not reimbursed for performing POCUS, why they have to cover the total expenses associated with POCUS by themselves [2, 12, 15]. Hence, in case POCUS is to be implemented in general practice a suggestion for a cost-covering remuneration can be useful for healthcare planners negotiating the fees for services. This leaves this study with two purposes: i) to investigate whether performing POCUS on patients with vaginal bleeding in early pregnancy in Danish general practices is cost-saving compared to usual practice; ii) to estimate the remuneration for Danish GPs performing POCUS on the respective patient population.

## Method

### Study design

A cost-minimisation analysis (CMA) was conducted to evaluate the cost-saving of performing POCUS in Danish general practices. The included patient population was pregnant women with vaginal bleeding in early pregnancy consulting their GP for the reassurance of a viable pregnancy. The CMA therefore focused on the two alternatives; GPs with access to POCUS and the ability to complete the clinical management of patients, and GPs without access to POCUS and therefore referring the patients to the secondary health care sector.

A prerequisite for applying a CMA is that the health outcomes between the compared alternatives demonstrate clinically equivalency [18, 19]. The CMA study design in this study was justified by the available evidence indicating a high level of inter-rater agreement for the ultrasonography scannings of intrauterine pregnancies performed by specialists and GPs, given GPs have had sufficient training [13]. In this type of analysis, only the costs between the alternatives are compared in order to identify the alternative with the lowest cost [18, 19]. The costs are considered from a societal perspective where all relevant costs are included, regardless of who bears the cost [20]. All costs are presented in 2019 prices and the prices were converted from Danish Kroner (DKK) into euros (€) using an exchange rate of DKK 7.47 = €1 [10.2019] [21].

The time horizon was defined as the duration of the clinical management of patients with vaginal bleeding in early pregnancy, starting with patients initially consulting the GP and ending at the last referred location, where a conclusive ultrasonography examination is performed. The duration of the clinical management is based on expert opinion and is typically within three days after consulting the GP. Due to this short time horizon, no discounting was applied.

The CMA was based on a decision analytical model constructed as a decision tree using the software programme TreeAge Pro 2019 v. R1.0 (TreeAge Software LCC, Williamstown, MA, USA).

### Model structure

**Fig. 1:**
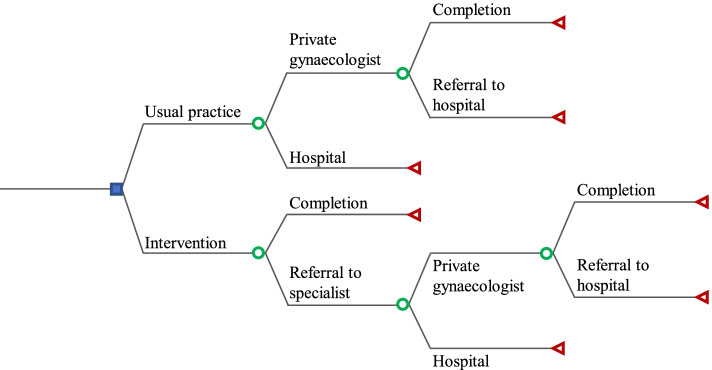
Structure of the decision tree for the usual practice and intervention

At the beginning of the decision tree, the decision node is illustrated as a square and represents the two alternatives: usual practice and intervention. Usual practice represents GPs without access to POCUS, while intervention represents GPs with access to POCUS. The subsequent branches are denoted with a chance node, illustrated as a circle, and represent the possible events in the pattern of referral for patients with vaginal bleeding in early pregnancy. Fig. 1 is a schematic representation of the model.

In the alternative representing usual practice, the possible events of the chance nodes are based on the guidelines for the clinical management of patients with vaginal bleeding in early pregnancy. The guidelines recommend that the GPs refer women with vaginal bleeding in early pregnancy to either a private gynaecologist or a gynaecological department at the hospital [14, 22, 23]. The private gynaecologist can either complete the management of the patient or the patient can be referred on to a hospital. At the hospital the clinical management of the patient will eventually be completed. By ‘*completion*’ it is understood that a conclusive examination and clinical management is performed, which is denoted with a terminal node, represented by a triangle.

In the alternative representing the intervention, the possible events are based on a study investigating GPs’ clinical management of patients when POCUS is accessible in Danish general practices. The GPs can either complete the clinical management of the patients by themselves or refer them to specialists. Referral to specialists is similar to usual practice and can be to either a private gynaecologist or a gynaecological department at the hospital. At the private gynaecologist, the patients can either be clinically managed or be further referred to the hospital. Thus in the intervention, the clinical management of the patients can be completed at the GP, the private gynaecologist, or at the gynaecological department in the hospital [24]. The completion is denoted with a terminal node.

### Model input parameters

The data in this study were derived from various sources, e.g. a self-developed questionnaire, Danish reports including guidelines, and expert opinions from clinical experts within the area of ultrasonography and gynaecology.

#### Probabilities

The probabilities of the different events in the model were derived from two sources including a questionnaire developed for the present study and a Danish report concerning modernisation of the medical field within gynaecology and obstetrics in Danish private practices. A questionnaire was developed for Danish GPs in order to investigate the pattern of referral for patients with vaginal bleeding in early pregnancy (See Additional file 1). The questionnaire was handed out to 22 Danish GPs at *the Danish Society for Ultrasonography in General Practice* annual conference in Denmark in 2019. Out of the 22 GPs, 21 GPs had access to POCUS in their clinical practice between 1–15 years and one GP did not have access. In the questionnaire, the GPs were asked to state how they would refer patients when having and not having access to POCUS in general practice. In addition, the GPs were asked to estimate the proportion of patients they would expect to be able to clinically manage when having access to POCUS. Based on the estimates from the GPs, average probabilities were calculated for the pattern of referral. (See Additional file 2).

In usual practice, 23% and 77% were used to express the likelihood of a patient being referred by the GP to a private gynaecologist and a hospital, respectively. In the intervention, the GPs expected to be able to complete 73% of the patients by themselves and refer the remaining 27% to a specialist. The conditional probabilities of a patient being referred to a private gynaecologist and a hospital in the intervention were estimated by the GPs to be 16% and 84%, respectively. After being referred to a private gynaecologist, patients can be exposed to two events in both alternatives, which are either a referral to the hospital or completion at the private gynaecologist. The probabilities of these two events are based on data from the Danish report concerning modernisation of the medical field within gynaecology and obstetrics in Danish private practices. The probability of patients being referred from a private gynaecologist to the hospital in both alternatives is 8%, and the probability for completion of clinical management at the private gynaecologist in both alternatives is 92% [23]. The model parameters for probabilities are shown in Table 1.

**Table 1:** Probabilities for the pattern of referral in base case

Pattern of referral	Probability	References
**Usual practice**		
Referral from GP to private gynaecologist	0.23	Questionnaire
Completion at private gynaecologist	0.92	[23]
Referral from private gynaecologist to hospital	0.08	[23]
Referral from GP to hospital	0.77	Questionnaire
**Intervention**		
Completion at GP	0.73	Questionnaire
Referral from GP	0.27	Questionnaire
Referral from GP to private gynaecologist	0.16	Questionnaire
Completion at private gynaecologist	0.92	[23]
Referral from private gynaecologist to hospital	0.08	[23]
Referral from GP to hospital	0.84	Questionnaire

#### Costs

The costs included in this study reflect the costs associated with the clinical management of patients with vaginal bleeding in early pregnancy. Only direct costs related to performing POCUS were considered in the base case analysis following a societal perspective. The costs are divided into four categories: ‘general practitioner’, ‘private gynaecologist’, ‘hospital’, and ‘patient’.

In the categories ‘general practitioner’, ‘private gynaecologist’, and ‘hospital’ the costs included for both alternatives are the consultation, which are based on national tariffs. The national tariffs for private gynaecologist and hospital include the costs for ultrasonography scanner, healthcare personnel wages, and utensils [25–27]. However, in the intervention, the average cost of GPs performing an ultrasonography scanning is added to the consultation tariff. This add-on cost reflects the cost of using the ultrasonography scanner, time consumption, GPs training in ultrasonography, and utensils used per ultrasonography scanning performed.

The cost of ultrasonography scanner and POCUS training are both estimated using depreciation [24, 28, 29]. The cost of having an ultrasonography scanner to examine the patients per scanning is calculated using an average price of an ultrasonography scanner of €15,508.70, the percentage of patients with vaginal bleeding in early pregnancy, an average lifespan of seven years, an interest rate of 4%, and an annual number of ultrasonography scannings of 2.4 per GP. The interest rate of 4% is defined by the Danish Ministry of Finance for socio-economic analyses, and the average lifespan of seven years is based on available literature on medical equipment [24, 28, 30].

In 2019, the ultrasonography training was provided by the Center of Clinical Ultrasound (CECLUS) in Denmark for €2,008. The CECLUS course consists of six categories: cardiovascular, lung, abdomen, gynaecology, musculoskeletal, skin. Within the category of gynaecology, there are four subcategories: intrauterine device, intrauterine foetus, heartbeat and CRL measurement. The cost of POCUS training to examine the patient population per scanning is calculated using the cost of CECLUS course for the subcategory heartbeat, an average lifespan of seven years, an interest rate of 4%, and the annual number of ultrasonography scannings of 2.4 per GP. The average lifespan of seven years is based on the assumption that GPs are expected to participate in an ultrasonography training course after seven years due to the development and renewal of medical equipment [21, 24, 31].

Based on the literature, the time consumption for performing POCUS is 10 min and the cost is based on a tariff [32]. The resource use of utensils per ultrasonography scanning is based on expert opinion and includes two wet wipes, one cover to the endocavitary transducer, and 10 ml gel. The unit costs for utensils are based on different sources from which GPs can purchase the utensils [33–35].

In the category for ‘patients’, the included costs for both alternatives consist of the consultation duration and the transportation cost in relation to time and distance. The resource use for patients is based on available literature, expert opinion, and unpublished data from the National Health Insurance Service Registry. In case no sources for the resource use were available (e.g. transportation time), assumptions were made.

In usual practice, a consultation at the GP lasts about 15 min, whereas in the intervention, the duration of the consultation is extended with 10 min, with a total of 25 min for consultation and ultrasonography examination [24]. Expert opinion indicates that the duration of the consultation is 25 min at the private gynaecologist and hospital for both alternatives. The unit cost is based on loss of earnings due to absence from work from “Amgros Estimating unit costs” and is €0.408 [36].

The transportation cost reflects the distance to each healthcare provider and transportation time. The average distance to the hospital is 19.6 km, and thus one visit at the hospital on average requires a distance of 39.2 km [37]. However, there is a lack of available information on the average distance to the GP and the private gynaecologist. Based on expert opinion, it is assumed that the average distance to a private gynaecologist is 19.6 km, and thus one visit at the private gynaecologist also will require 39.2 km on average. In addition, based on the unpublished data from The National Health Insurance Service Registry, the average distance to the GPs is estimated to be 4.75 km each way, or 9.5 km for one visit. The unit cost of the travel distances is based on compensation from “Amgros Estimating unit costs” and is €0.473 per kilometre [36].

To calculate the transportation time, it is assumed that patients are travelling by car with an average speed of 50 km per hour (km/h). The distance to each location is divided by the average speed. In total, the distance to GP, private gynaecologist, and hospital is covered in 11 min, 47 min, and 47 min, respectively. The unit cost is based on loss of earnings due to absence from work from “Amgros Estimating unit costs” and is €0.408 [36]. The resource use and unit price for the four categories are listed in Table 2.

**Table 2:** Cost parameters for the identified categories

Identified cost parameters	Resource use	Unit price (€)	References
**General practitioner**			
Tariff for consultation	1	19.02	[26]
Time consumption for performing ultrasonography	10 min	1.807	[32]
Ultrasonography scanner	1	7.54	[24]
CECLUS	1	5.81	[24]
Utensils			
Wet wipes	2	0.054	[35]
Transducer cover	1	0.24	[33]
Gel	10 ml	0.021	[34]
**Private gynaecologist**			
Tariff for consultation with ultrasonography	1	96.78	[27]
**Hospital**			
DRG-tariff for performing ultrasonography^a^	1	164.26	[25]
**Patients**			
Patient time			
General practitioner without ultrasonography	15 min	0.408	[24, 36]
General practitioner with ultrasonography	25 min	0.408	[24, 36]
Private gynaecologist	25 min	0.408	[24, 36]
Hospital	25 min	0.408	[24, 36]
Transportation distance			
General practitioner	9.5 km	0.473	[36, 37]
Private gynaecologist	39.2 km	0.473	[36, 37]
Hospital	39.2 km	0.473	[36, 37]
Transport time			
General practitioner	11 min	0.408	[36]
Private gynaecologist	47 min	0.408	[36]
Hospital	47 min	0.408	[36]

^a^DRG-tariff is based on a combination of a principal diagnosis code for patients with bleeding in early pregnancy (DO209), and the procedure code for transvaginal ultrasonography of female genitals (UXUD82).

### Decision rule

Once the costs and probabilities were assigned to each event, the costs were multiplied by their respective probabilities and summed across the nodes within a particular branch. The average cost of the branches of each particular alternative: usual practice and intervention, was subsequently estimated to decide which alternative was the most favourable. In this CMA, the most favourable alternative was the one with the lowest cost as this reflects the most cost-saving alternative for the Danish society. Following the calculation of the average cost of both alternatives, the incremental cost was found. The incremental cost reflects the difference in the two alternatives and is calculated by subtracting the average cost of usual practice from the average cost of the intervention.

### Sensitivity analysis

To check the robustness of the base case results, deterministic one-way sensitivity analyses and a scenario analysis were conducted. Analyses were performed by varying one parameter at a time within a plausible range, while holding the others constant, thereby, estimating the impact of each parameter on the incremental cost [38].

The probability parameters for the pattern of referral and the cost parameters: ultrasonography, utensils, CECLUS training, consultation cost at the GP, private gynaecologist, hospital, the patient consultation time, transportation distance, and transportation time were analysed. The ranges for probability parameters were based on unpublished data from the questionnaire developed for this study, in which the ranges were defined as the lowest and highest stated probability in the questionnaire. The ranges for consultation cost at the GP, private gynaecologist, and hospital were defined based on the annual percentage increase in consultation tariff [25–27, 39–41]. The range for the cost of ultrasonography scanner is based on data of prices of ultrasonography scanners at GPs in Denmark [24]. The range for the costs of utensils used per ultrasonography scan at the general practitioner is based on expert opinion. The range for the costs of an ultrasonography course is based on the courses offered in Denmark. Aside from the CECLUS course, a course is offered in cooperation with the Danish Society of Diagnostic Ultrasound [42]. This course consists of five areas, with one of the areas being early pregnancy complications. The calculation method for estimating the annual depreciation cost of this course was similar to the CECLUS course.

#### Patient-related costs

The range for patients’ time spent on consultation with the GP is based on available literature [24]. As there is a lack of evidence on data for the consultation time at the specialists, it is assumed that the patients’ consultation time at the private gynaecologist and hospital can vary within the same ranges as a consultation in the intervention where GPs perform ultrasonography.

The ranges for the distance to the GP and the specialists is based on The National Health Insurance Service Registry and an analysis from Local Government Denmark, respectively. The ranges for transportation time are changed based on the ranges for the distance [37]. The ranges used for varying the model parameters for probabilities and costs are listed in Table 3.

**Table 3:** Model parameters for probabilities and costs

Parameters	Ranges	References
**Probabilities**		
**Usual practice**		
Referral from GP to private gynaecologist	[0.00 – 1.00]	Questionnaire
Completion at private gynaecologist	[0.92 – 1.00]	[23]
Referral from private gynaecologist to hospital	[0.00 – 0.08]	[23]
Referral from GP to hospital	[0.00 – 1.00]	Questionnaire
**Intervention**		
Completion at GP	[0.40 – 0.98]	Questionnaire
Referral from GP	[0.02 – 0.60]	Questionnaire
Referral from GP to private gynaecologist	[0.00 – 1.00]	Questionnaire
Completion at private gynaecologist	[0.92 – 1.00]	[23]
Referral from private gynaecologist to hospital	[0.00 – 0.08]	[23]
Referral from GP to hospital	[0.00 – 1.00]	Questionnaire
**Costs**		
**General practitioner**		
Cost of ultrasonography scanner	[1.30 – 13.77]	[24]
Cost of consultation without ultrasonography	[19.02 – 19.25]	[39, 40]
Cost of ultrasonography training	[3.35 – 5.81]	[31, 42]
Cost of utensils	[0.34 – 0.56]	Expert opinion
**Private gynaecologist**		
Cost of consultation	[96.78 – 97.99]	[27, 41]
**Hospital**		
Cost of consultation	[164.26 – 164.79]	[25]
**Patient-related parameters**		
**Patients**		
Consultation time		
General practitioner without ultrasonography	[5-15]	[24]
General practitioner with ultrasonography	[13-26]	[24]
Private gynaecologist	[13-26]	[24]
Hospital	[13-26]	[24]
Transportation distance		
General practitioner	[0.2—42]	Unpublished registry data
Private gynaecologist	[0.2—100]	[37]
Hospital	[0.2 – 100]	[37]
Transportation time		
General practitioner	[0.24 – 50.40]	Unpublished registry data
Private gynaecologist	[0.24 – 120]	[37]
Hospital	[0.24 – 120]	[37]

Additionally, a scenario analysis was conducted in which the study perspective was changed from societal to a healthcare sector perspective. In a healthcare sector perspective, only the costs borne by the healthcare sector are included, thus excluding the patient-related costs [20].

Results from the deterministic one-way sensitivity analyses are illustrated in a tornado diagram. Here, the bars in the diagram are arranged in order with the widest bar representing the most influential parameter at the top of the tornado and the least influential at the bottom [29, 43].

A probabilistic sensitivity analysis was performed based on a second order Monte Carlo simulation with 10,000 simulations to investigate the impact of multivariate uncertainty on the model results [18]. In this sensitivity analysis, all input parameters, excluding unit costs for mileage compensation, GP’s time consumption for performing ultrasonography, and patient’s time consumption, were included with the following assigned distributions: beta distribution for probability parameters and gamma distribution for cost parameters.

### Remuneration

In different countries including Denmark and the UK, remuneration is used to influence the activities of GPs [44].

The remunerations for the services GPs perform reflect the total cost of the resource use for the particular service. Currently, in Danish general practices GPs are not reimbursed for performing POCUS and POCUS is usually not an integrated part of the consultation [12]. In order to allocate this service from secondary to primary healthcare sector, GPs must be reimbursed for this service. The remuneration proposed in this study is the add-on cost of Danish GPs performing POCUS on patients with vaginal bleeding in early pregnancy. This add-on cost consists of the following cost components: cost of ultrasonography scanner, cost of ultrasonography training, cost of utensils per scanning, and cost of time consumption for performing ultrasonography.

## Results

### Cost-minimisation analysis

From a societal perspective, the average costs for performing POCUS on patients with vaginal bleeding in early pregnancy is €125 per patient in the intervention. In usual practice, where the patient population is referred to the private gynaecologist or/and hospital for an ultrasonography scan the average cost is €235 per patient. This indicates that having access and the ability to perform POCUS in general practice on patients with vaginal bleeding in early pregnancy from a societal perspective is favourable, resulting in an average cost-saving of €110 per patient. The costs associated with each alternative are shown in Table 4.

**Table 4:** Costs associated with usual practice and intervention

Costs in each alternative	Usual practice (€)	Intervention (€)	References
**General practitioner**			
Tariff for consultation	19.02	19.02	[26]
Time consumption for performing ultrasonography	-	18.07	[32]
Ultrasonography scanner	-	7.54	[24]
CECLUS	-	5.81	[24]
Utensils			
Wet wipes	-	0.108	[35]
Transducer cover	-	0.24	[33]
Gel	-	0.21	[34]
**Private gynaecologist**			
Tariff for consultation with ultrasonography	96.78	96.78	[27]
**Hospital**			
DRG-tariff for performing ultrasonography^a^	164.26	164.26	[25]
**Patients**			
Patient time			
General practitioner without ultrasonography	6.12	-	[24, 36]
General practitioner with ultrasonography	-	10.2	[24, 36]
Private gynaecologist	10.2	10.2	[24, 36]
Hospital	10.2	10.2	[24, 36]
Transportation distance			
General practitioner	4.49	4.49	[36, 37]
Private gynaecologist	18.5	18.5	[36, 37]
Hospital	18.5	18.5	[36, 37]
Transport time			
General practitioner	4.49	4.49	[36]
Private gynaecologist	19.18	19.18	[36]
Hospital	19.18	19.18	[36]

^a^DRG-tariff is based on a combination of a principal diagnosis code for patients with bleeding in early pregnancy (DO209), and the procedure code for transvaginal ultrasonography of female genitals (UXUD82).

### Sensitivity analysis

**Fig. 2:**
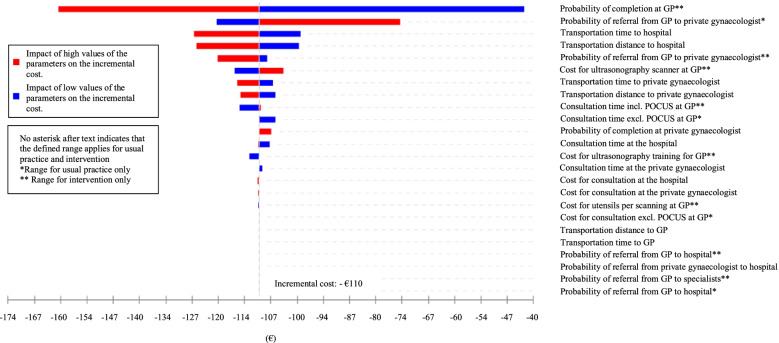
Tornado diagram of the base case analysis

The results from the deterministic sensitivity analysis are depicted in a tornado diagram shown in Fig. 2. The bars for all of the parameters remain within a negative incremental cost, and the results from the sensitivity analyses thus demonstrate the robustness of the base case result to plausible changes in all of the input parameters. The parameter representing the probability of completing the clinical management of the patient population at the GP is the most influential as changes in this parameter have the largest influence on the incremental cost.

The change of study perspective to a healthcare sector perspective, i.e. exclusion of patient-related costs, resulted in an incremental cost-saving of €78 per patient. This indicates that the incremental cost is affected by being less cost-saving compared to the base case analysis, but the patient-related costs do not have a decisive impact on the result as the intervention remains favourable.

**Fig. 3:**
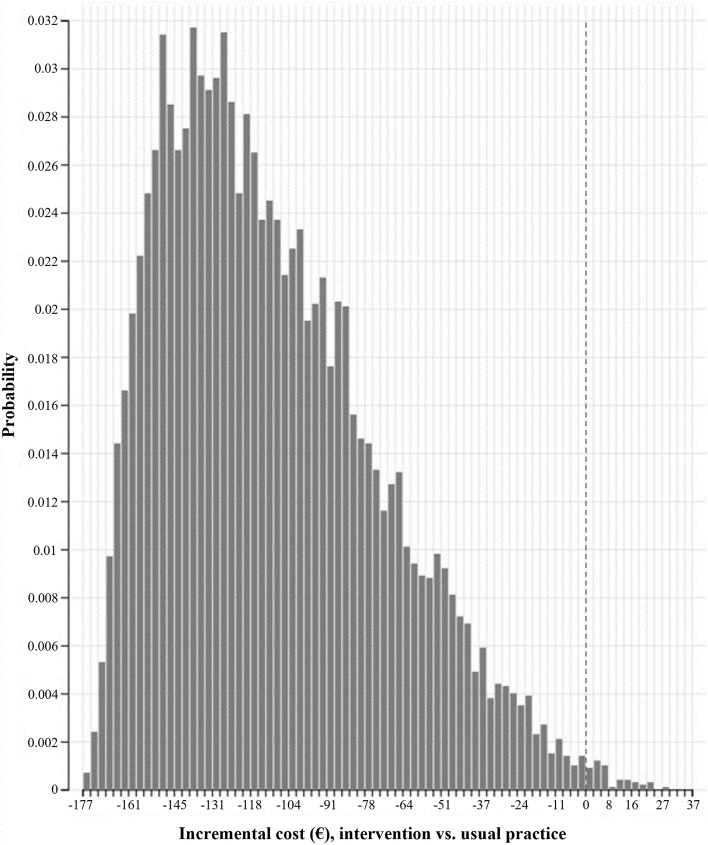
Distribution of the incremental cost following Monte Carlo simulation

Monte Carlo simulation indicated that the intervention was cost-saving compared to usual practice in majority of the simulations in which the incremental cost was close to the base case result of €110. The distribution of the incremental cost following the Monte Carlo simulation is shown in Fig. 3.

### Estimated remuneration

The remuneration is estimated based on the identified costs from the base case analysis in the CMA that is directly associated with GPs performing POCUS in general practice. The add-on cost of performing POCUS on patients with vaginal bleeding in early pregnancy was calculated to be €32. This add-on cost was assigned to the costs related to the GP in the alternative for the intervention and indicated cost-saving compared to the alternative reflecting usual practice. The cost components of the remuneration are shown in Table 5.

**Table 5:** Estimated remuneration for GPs performing POCUS on the respective patient population

Identified cost components	Resource use	Unit price (€)	Cost (€)	References
**Time consumption for performing ultrasonography**	10 min	1.807	18.07	[32]
Ultrasonography scanner	1	7.54	7.54	[24]
CECLUS	1	5.81	5.81	[31]
Utensils				
Wet wipes	2	0.054	0.108	[35]
Transducer cover	1	0.24	0.24	[33]
Gel	10 ml	0.021	0.21	[34]
**Total**			**31.98**	

## Discussion

### Study findings

This study showed that performing POCUS in general practice on patients with vaginal bleeding in early pregnancy is estimated to be cost-saving with an average saving of €110 per patient compared to usual practice. The deterministic sensitivity analyses revealed that regardless of plausible changes in the input parameters and study perspective, the model was robust as the intervention remained cost-saving. The robustness of the findings was further investigated using a probabilistic sensitivity analysis. This analysis demonstrated that the intervention compared to usual practice was cost-saving with a high degree of certainty.

The remuneration for GPs performing POCUS was estimated to be €32, reflecting the costs associated with an ultrasonography scanner, GP’s time consumption, ultrasonography training, and utensils per ultrasonography scanning performed.

### Strengths and limitations

The cost-minimisation analysis, assuming an equivalent ultrasonography scanning quality between GP and specialists, swiftly allowed the potential benefits in terms of cost-savings related to performing POCUS in general practice to be identified. This economic approach to decision-making that solely focuses on the costs is advantageous as it is simple to conduct. However, the simplicity of a CMA compared to the traditional full economic evaluations has made its application in decision-making questionable as the available literature indicates scepticism around CMA as a full economic evaluation [45, 46]. In an article by Briggs et al., 2001 [45], it is pointed out that there is an uncertainty around the estimates of costs and effects and it is due to this uncertainty that the use of CMA for decision-making is questionable. Thus, the use of CMA for decision-making in this study may be a limitation and it may be preferable to collect and analyse data on health outcomes to assess this uncertainty. However, according to available evidence, the theoretical basis of CMA must be acknowledged to be as rigorous as the theoretical basis of other economic evaluations [46].

In a prospective study by Lindgaard et al., 2017 [13], the diagnostic agreement between GPs and specialists performing POCUS for intrauterine pregnancy was evaluated. The GPs included in the study were enrolled in the CECLUS course and the findings of this study showed that GPs performing POCUS with low-moderate complexity had a high inter-rater agreement compared to specialists. As the available literature indicates equivalency in the quality of POCUS scannings (Cohen’s kappa value = 1, almost perfect agreement) between the GPs and specialists, CMA is believed to be an appropriate study approach for the present economic evaluation. [13].

The strength of applying a decision analytical framework for this study’s purposes is that it allows synthesising all available and relevant evidence from multiple sources, including expert opinions, registries, and reports. The use of decision analysis also enables decision-makers to identify gaps in the current evidence and clarify the need for and value of more evidence which can be beneficial for future decision-making. As this study is the first of its kind to investigate the cost-saving of performing POCUS on patients with vaginal bleeding in early pregnancy, it is beneficial to use decision analysis to clarify the need for more evidence. [47, 48].

The main limitation of this study is the model being based on very early modelling with low-quality evidence. Firstly, the probabilities of referral from the private gynaecologist to the hospital and the probability of completing the clinical management at the private gynaecologist are based on a Danish report [23]. The report on the pattern of referral from private specialists to the hospital applies for several medical areas and is non-specific. Therefore, it is unknown how well these data apply to the patient population in this study.

Secondly, the use of non-empirical parameter estimates based on expert opinions is a limitation as it may lead to over- and underestimations. To reflect real-life, the referral probabilities in the decision tree were based on unpublished data from a questionnaire developed for this study. However, these data are not validated in a clinical setting and the data precision is unknown. [49] Moreover, using the opinion of Danish experts may hinder the generalizability of the study findings to other settings, where the healthcare system and the patient pathway for the respective patient population differ from the Danish setting. Due to these limitations, the authors of this paper suggest the model to be refined to a full economic evaluation when new evidence is available.

### Comparison to existing literature

To the best of our knowledge, presently, no other published studies have been identified investigating the possible cost-savings associated with performing POCUS in general practice on patients with vaginal bleeding in early pregnancy. However, Wordsworth et al., 2002 [50] investigated the costs of performing ultrasonography scannings in general practice compared to the hospital. Scanning in general practice resulted in a reduction in total costs per year with reduced out-patient referrals. Although Wordsworth et al., 2002 [50] does not specifically include patients with vaginal bleeding in early pregnancy, it reflects a similar healthcare setting and thus supports the advantages of having access to POCUS in general practice. Moreover, the reduced out-patients referrals shown in Wordsworth et al., 2002 [50] is also seen in the present study as the results from the questionnaires investigating the GPs pattern of referral indicated reduced possible referrals to specialists when having access to POCUS in general practice.

In an article by Colli et al., 2015 [51], the pattern of referral, when GPs have access to POCUS was investigated. This study used different patient populations than patients within the area of gynaecology/obstetrics, but still supports that POCUS in general practice and simple training of GPs can reduce the number of referrals and tests, thereby leading to healthcare savings [51].

### Implication for future research

In this study, equivalency in the health outcome, quality of ultrasonography scannings, was assumed based on the available evidence. However, there may be other health outcomes that are clinically meaningful to patients with vaginal bleeding in early pregnancy, which can demonstrate additional value of the intervention compared to usual practice. Data collection of health outcomes such as patient satisfaction or patient preferences can be preferable to demonstrate the patient’s point of view. For this reason, future research on among others these health outcomes can be used to refine the current model.

## Conclusion

This study found that the use of POCUS in general practice for patients with vaginal bleeding in early pregnancy is cost-saving based on the currently available evidence. This study is based on early modelling, why additional evidence is required to refine the model and support healthcare decision-making.

In addition to the CMA, the expected remuneration for GPs performing POCUS on the respective patient population was estimated. Such calculation may qualify negotiation regarding remuneration for such services.

## Supplementary Information

**Additional file 1.** Template for questionnaire

**Additional file 2.** Raw response data from anonymous general practitioners

## Data Availability

The datasets used and/or analysed during the current study are available from the corresponding author on reasonable request.

## Abbreviations

DKK: Danish Kroner
CECLUS: Center of Clinical Ultrasound
CMA: Cost-minimisation analysis
GP: General practitioner
POCUS: Point-of-care ultrasonography
UK: United Kingdom

## References

[1] Weile J, Brix J, Moellekaer AB (2018). Is point-of-care ultrasound disruptive innovation? Formulating why POCUS is different from conventional comprehensive ultrasound. Crit Ultrasound J.

[2] Moore CL, Copel JA (2011). Point-of-Care Ultrasonography. N Engl J Med.

[3] Soni NJ, Schnobrich D, Mathews BK, Tierney DM, Jensen TP, Dancel R (2019). Point-of-Care Ultrasound for Hospitalists: A Position Statement of the Society of Hospital Medicine. J Hosp Med..

[4] Abu-Zidan FM, Cevik AA (2018). Diagnostic point-of-care ultrasound (POCUS) for gastrointestinal pathology: State of the art from basics to advanced, World Journal of Emergency Surgery. BioMed Central Ltd.

[5] Genc A, Ryk M, Suwała M, Żurakowska T, Kosiak W (2016). Ultrasound imaging in the general practitioner’s office - a literature review. J Ultrason.

[6] Kimura B, DeMaria A (2008). The, “Laying on” of ultrasound. JACC Cardiovasc Imaging.

[7] Bhagra A, Tierney D, Sekiguchi H (2016). Point-of-care ultrasonography for primary care physicians and general internists. Mayo Clin Proc.

[8] Implementation of clinical ultrasound in general practice [Internet]. 2019 [cited 2021 Mar 13]. Available from: http://www.apo-danmark.dk/files/pub/5432.pdf

[9] Breeze C. Early pregnancy bleeding. R Aust Coll Gen Pract. 2016;45(5). 27166462

[10] Strommen J, Masullo L, Crowell T, Moffett P (2017). First–trimester vaginal bleeding: Patient expectations when presenting to the emergency department. Mil Med.

[11] Andersen CA, Jensen MBB, Toftegaard BS, Vedsted P, Harris M, Research Group Ö (2019). Primary care physicians’ access to in-house ultrasound examinations across Europe: A questionnaire study. BMJ Open.

[12] Mengel-Jørgensen T, Jensen MB (2016). Variation in the use of point-of-care ultrasound in general practice in various European countries. Results of a survey among experts. Eur J Gen Pract. Taylor and Francis Ltd.

[13] Lindgaard K, Riisgaard L (2017). Validation of ultrasound examinations performed by general practitioners. Scand J Prim Health Care.

[14] Recommendations for antenatal care. Sundhedsstyrelsen; 2013.

[15] Andersen CA, Holden S, Vela J, Rathleff MS, Jensen MB (2019). Point-of-care ultrasound in general practice: A systematic review. Ann Fam Med.

[16] Bornemann P, Jayasekera N, Bergman K, Ramos M, Gerhart J (2018). Point-of-care ultrasound: Coming soon to primary care?. J Fam Pr.

[17] Pedersen KM, Andersen JS, Snødergaard J (2012). General practice and primary health care in Denmark. J Am Board Fam Med.

[18] Rudmik L, Drummond M (2013). Health economic evaluation: Important principles and methodology. Laryngoscope..

[19] Haycox MA. What is cost-minimisation analysis? [Internet]. 2009 [cited 2021 Mar 13]. Available from: http://www.bandolier.org.uk/painres/download/whatis/What_is_cost-min.pdf

[20] Ehlers LH, Soerensen AS (2019). Costing in health economic evaluation: Theory and practice.

[21] National Bank of Denmark: Exchange rates [Internet]. [cited 2021 Mar 13]. Available from: https://nationalbanken.statistikbank.dk/nbf/107312

[22] Report on the specialty: Gynaecology and Obstetrics. 2017 [cited 2021 Mar 13]; Available from: https://www.sst.dk/-/media/Viden/Specialplaner/Specialeplan-for-gynækologi-og-obstetrik/Specialerapport-for-Gynaekologi-og-obstetrik.ashx?la=da&hash=E887B2593F0AB29707CFCE5B6804EC3ED28F0AF4

[23] Report on modernisation of the medical field within gynaecology and obstetrics [Internet]. 2018 [cited 2021 Mar 13]. Available from: https://www.regioner.dk/media/10948/moderniseringsrapport-gynaekologi-revideret-29-november-2018.pdf

[24] Aakjær Andersen C, Brodersen J, Davidsen AS, Graumann O, Jensen MBB (2020). Use and impact of point-of-care ultrasonography in general practice: A prospective observational study. BMJ Open..

[25] Interactive DRG database [Internet]. [cited 2019 Oct 19]. Available from: https://interaktivdrg.sundhedsdata.dk/#/

[26] Tariffs for general practitioners [Internet]. 2019 [cited 2021 Mar 13]. Available from: https://www.laeger.dk/sites/default/files/honorartabel_2019_april_web.pdf

[27] Tariffs for gynaecology and obstetrics [Internet]. 2019 [cited 2021 Mar 13]. Available from: https://www.laeger.dk/sites/default/files/takstkort_-_gynaekologi_pr._01._oktober_2018.pdf

[28] Interest rate for health economic analyses [Internet]. [cited 2021 Mar 13]. Available from: https://fm.dk/nyheder/nyhedsarkiv/2013/maj/ny-og-lavere-samfundsoekonomisk-diskonteringsrente/

[29] Drummond MF. Methods for the Economic Evaluation of Health Care Programmes. Oxford University Press; 2015.

[30] Average lifespan of ultrasonography scanner [Internet]. [cited 2021 Mar 13]. Available from: https://www.medicaldevicedepot.com/Articles.asp?ID=307

[31] Center of Clinical Ultrasound (CECLUS) - Course [Internet]. [cited 2021 Mar 13]. Available from: https://health.au.dk/uddannelse/efter-og-videreuddannelse/kurser/ultralyd/

[32] Tariff for data collection [Internet]. [cited 2019 Oct 19]. Available from: https://www.dsam.dk/forskning/multipraksisudvalget/honorar_for_dataindsamling_til_praktiserende_laeger/

[33] Cost of cover to endocavitary transducer [Internet]. [cited 2019 Oct 19]. Available from: https://mediqdanmark.dk/katalog/diagnostik/dopplereultralydsgelogovertraek/1637/6070130

[34] Cost for gel [Internet]. [cited 2019 Oct 19]. Available from: https://www.med24.dk/medicin-og-medicare/hjemmetest/hjertelydsmonitor/ultralydsgel-250ml

[35] Cost for wet wipes [Internet]. [cited 2019 Oct 19]. Available from: https://haandsprit.dk/overfladedesinfektionsservietter

[36] Amgros estimating unit costs [Internet]. [cited 2021 Mar 13]. Available from: https://www.amgros.dk/media/2223/amgros-vaerdisaetning-af-enhedsomkostninger.pdf

[37] Hansen B, Toft L. Distance to public hospitals in Denmark [Internet]. 2016 [cited 2019 Oct 19]. Available from: https://www.kl.dk/media/18668/afstand-til-naermeste-sygehus-fugleflugt-eller-vejafstand.pdf

[38] Cairns J, Fox-Rushby J. Economic Evaluation. Open University Press; 2005.

[39] Tariffs for general practitioners [Internet]. 2015 [cited 2019 Oct 19]. Available from: www.laeger.dk

[40] Tariffs for general practitioners [Internet]. 2020 [cited 2019 Oct 19]. Available from: https://www.laeger.dk/sites/default/files/honorartabel_2019_oktober-1.pdf

[41] Tariffs for medical specialists [Internet]. 2017 [cited 2019 Oct 19]. Available from: https://rn.dk/sundhed/til-sundhedsfaglige-og-samarbejdspartnere/sundhedsfaglige-raad-og-udvalg/samarbejdsudvalg-for-speciallaeger/-/media/Rn_dk/Sundhed/Til-sundhedsfaglige-og-samarbejdspartnere/Sundhedsfaglige-raad-og-udvalg/Samarbejds-og-koordinationsudvalg/Samarbejdsudvalg-for-speciallaeger/Overenskomst-speciallaege.ashx

[42] Danish Society of Diagnostic Ultrasound - Course [Internet]. [cited 2021 Mar 13]. Available from: https://www.drejergaarden.dk/kurser/ultralydkursus.php

[43] Taylor M. What is sensitivity analysis? [Internet]. 2009 [cited 2021 Mar 13]. Available from: http://www.bandolier.org.uk/painres/download/What is 2009/What_is_sens_analy.pdf

[44] New structure of tariffs in general practice [Internet]. 2012 [cited 2021 Mar 13]. Available from: https://www.vive.dk/media/pure/8573/2035606

[45] Briggs AH, O’Brien BJ (2001). The death of cost-minimization analysis?. Heal Econ.

[46] Vohora D, Singh G (2017). Pharmaceutical Medicine and Translational Clinical Research.

[47] Petrou S, Gray A (2011). Economic evaluation using decision analytical modelling: Design, conduct, analysis, and reporting. BMJ.

[48] Grutters JPC, Joore MA, Van Der Horst F, Stokroos RJ, Anteunis LJC (2008). Decision-analytic modeling to assist decision making in organizational innovation: The case of shared care in hearing aid provision. Health Serv Res..

[49] Kvale S, Brinkmann S. InterViews - Learning the Craft of qualitative Research Interviewing. Hans Reitzel; 2015.

[50] Wordsworth S, Scott A (2002). Ultrasound scanning by general practitioners: Is it worthwhile?. J Public Heal Med.

[51] Colli A, Prati D, Fraquelli M, Segato S, Vescovi PP, Colombo F (2015). The use of a pocket-sized ultrasound device improves physical examination: Results of an in- and outpatient cohort study. PLoS One..

