# Supplementary material for: A cost-minimisation analysis of performing point-of-care ultrasonography on patients with vaginal bleeding in early pregnancy in general practice: a decision analytical model
Source: BMC Health Serv Res. 2022 Jan 11;22:55. doi: 10.1186/s12913-022-07463-y (PMC8753911; doi:10.1186/s12913-022-07463-y)
Supplement: Supplementary file 1 — Additional file 1. Template for questionnaire [file 12913_2022_7463_MOESM1_ESM.pdf]

Additional file 1. Template for questionnaire.

(translated from the original Danish version)

Questionnaire regarding patients with vaginal bleeding in early pregnancy who consult GPs

This questionnaire is developed to GPs who see the patient population: *Women with vaginal bleeding in early pregnancy (1st trimester)*. The purpose of this questionnaire is to investigate the pattern of referral for this patient population in the two different scenarios: scenario A and scenario B. The scenarios vary in relation to having access to an ultrasonography scanner in the consultation with the patient. ***Answers to both scenarios are required!***

Gender: ☐ Female ☐ Male ☐ Other

Work location: ☐ North Denmark Region ☐ Central Denmark Region ☐ Region of Southern Denmark ☐ Region Zealand ☐ Capital Region of Denmark

Do you use ultrasonography scanner in your daily work? ☐ Yes ☐ No

If yes, how many years have you used an ultrasonography scanner? ________ Year(s)

Scenario A: You do not have access to an ultrasonography scanner

**A.1:** If you DO NOT have the possibility to perform a transvaginal ultrasonography scanning in your consultation with women with vaginal bleeding in early pregnancy (1st trimester), where will you then refer these patients to:	☐ Mostly/only private gynaecologist<br>☐ Mostly/only hospital<br>☐ It varies, I refer to both<br>☐ None of the above (please elaborate on the back of the paper)
**A.2:** If it varies, what percentage of the patients will you refer to a private gynaecologist or to the hospital, respectively?	Private gynaecologist: ______________%<br>Hospital: ______________%

Scenario B: You do have access to an ultrasonography scanner

**B.1:** What percentage of women with vaginal bleeding in early pregnancy (1st trimester) do you estimate to complete the clinical management of?	______________%
**B.2:** If you think you are not able to complete the clinical management of the patient, where will you then refer the patients to?	☐ Mostly/only private gynaecologist<br>☐ Mostly/only hospital<br>☐ It varies, I refer to both<br>☐ None of the above (please elaborate on the back of the paper)
**B.3:** If it varies, what percentage of the patients that you are not able to complete the clinically management of, will you refer to a private gynaecologist or to the hospital, respectively?	Private gynaecologist: ______________%<br>Hospital: ______________%

Elaboration of question A.1

Elaboration of question B.2

Additional comments

Thank you for your response!

FORSKNINGSENHEDEN FOR ALMEN PRAKSIS
Aalborg

AALBORG UNIVERSITET
