# Supplementary material for: A cost-minimisation analysis of performing point-of-care ultrasonography on patients with vaginal bleeding in early pregnancy in general practice: a decision analytical model
Source: BMC Health Serv Res. 2022 Jan 11;22:55. doi: 10.1186/s12913-022-07463-y (PMC8753911; doi:10.1186/s12913-022-07463-y)
Supplement: Supplementary file 2 — Additional file 2. Raw response data from anonymous general practitioners [file 12913_2022_7463_MOESM2_ESM.pdf]

# Additional file 2. Raw response data from anonymous general practitioners.

| Doctor No. | Gender | Region | POCUS in usual practice | Year(s) | A.1 | A.2 (%, gynaecologist/hospital) | B.1 (%) | Referral (%) | B.2 | B.3 (%, gynaecologist/hospital) | Additional comments |
|---|---|---|---|---|---|---|---|---|---|---|---|
| 1 | Female | Central Denmark Region | No | - | Hospital | 0/100 | 50 | 50 | Hospital | 0/100 | - |
| 2 | Male | Region of Southern Denmark | Yes | 10 | Hospital | 0/100 | 60 | 40 | Hospital | 0/100 | - |
| 3 | Male | Central Denmark Region | Yes | 2 | Hospital | 0/100 | 50 | 50 | Hospital | 0/100 | - |
| 4 | Male | North Denmark Region | Yes | 5 | Hospital | 0/100 | 75 | 25 | Hospital | 0/100 | - |
| 5 | - | Capital Region of Denmark | Yes | - | Hospital | 0/100 | 40 | 60 | Hospital | 0/100 | - |
| 6 | Female | Central Denmark Region | Yes | 15 | Hospital | 1/99 | 92,5 | 7,5 | Hospital | 0/100 | - |
| 7 | Male | Region of Southern Denmark | Yes | 5 | Private gynaecologist | 100/0 | 95 | 5 | Private gynaecologist | 100/0 | B.2: only when treatment at the hospital is expected to be required. |
| 8 | Male | North Denmark Region | Yes | 6 | Hospital | 0/100 | 95 | 5 | Hospital | 0/100 | B.2: Often I can complete the clinical management of the patients. In case of suspicion of extrauterine pregnancy, patients are referred. |
| 9 | Female | Region of Southern Denmark | Yes | 1 | Hospital | 0/100 | 80 | 20 | Hospital | 0/100 | A.1: The number of private gynaecologists in my area is very limited. |
| 10 | Male | Capital Region of Denmark | Yes | 2 | Hospital | 0/100 | 95 | 5 | Hospital | 0/100 | - |
| 11 | Male | Region of Southern Denmark | Yes | 2,5 | - | - | 50 | 50 | Hospital | 0/100 | - |
| 12 | Male | North Denmark Region | Yes | 4 | Hospital | 20/80 | 80 | 20 | Hospital | 10/90 | B.2: Patients who are worried about spontaneous abortion are very happy to have a fast clarification without the need for a referral. |
| 13 | Male | North Denmark Region | Yes | 4 | Private gynaecologist | 90/10 | 80 | 20 | Both | 30/70 | A.1: Hospitals offer acute consultations. Other: Often hospital as further examinations or operations are often required. |
| 14 | Male | Region of Southern Denmark | Yes | 2 | Both | 30/70 | 75 | 25 | Private gynaecologist | 50/50 | - |
| 15 | Male | Region of Southern Denmark | Yes | 1 | Both | 75/25 | 75 | 25 | Both | 50/50 | - |
| 16 | Male | Central Denmark Region | Yes | 2 | Hospital | 10/90 | 50 | 50 | Hospital | 10/90 | - |
| 17 | Female | North Denmark Region | Yes | 4 | Hospital | 0/100 | 60 | 40 | Hospital | 0/100 | - |
| 18 | Male | Region of Southern Denmark | Yes | 5 | Hospital | 0/100 | 90 | 10 | Hospital | 20/80 | - |
| 19 | Male | Region of Southern Denmark | Yes | 5 | Private gynaecologist | 100/0 | 98 | 2 | Hospital | 0/100 | B.2: If patients have a missed abortion, I refer them to the hospital for a curettage of the uterus. |
| 20 | Male | Capital Region of Denmark | Yes | 6 | Both | 25/75 | 66 | 34 | Both | 10/90 | - |
| 21 | Male | Capital Region of Denmark | Yes | 1 | Hospital | 10/90 | 75 | 25 | Hospital | 0/100 | - |
| 22 | Male | Capital Region of Denmark | Yes | 3 | - | - | 75 | 25 | Both | 75/25 | - |
| **Average** | | | | **3,43** | | **23/77** | **73,02** | **26,98** | | **16/84** | |

The sign (-) indicates no response.
